# Charging OBO‐Fused Double [5]Helicene with Electrons

**DOI:** 10.1002/anie.201908658

**Published:** 2019-09-09

**Authors:** Zheng Zhou, Xiao‐Ye Wang, Zheng Wei, Klaus Müllen, Marina A. Petrukhina

**Affiliations:** ^1^ Department of Chemistry University at Albany, State University of New York 1400 Washington Ave. Albany NY 12222 USA; ^2^ Max Planck Institute for Polymer Research Ackermannweg 10 55128 Mainz Germany

**Keywords:** alkali metals, chemical reduction, helicene, NMR spectroscopy, X-ray diffraction

## Abstract

Chemical reduction of OBO‐fused double[5]helicene with Group 1 metals (Na and K) has been investigated for the first time. Two doubly‐reduced products have been isolated and structurally characterized by single‐crystal X‐ray diffraction, revealing a solvent‐separated ion triplet (SSIT) with Na^+^ ions and a contact‐ion pair (CIP) with K^+^ ion. As the key structural outcome, the X‐ray crystallographic analysis discloses the consequences of adding two electrons to the double helicene core in the SSIT without metal binding and reveals the preferential binding site in the CIP with K^+^ counterions. In both products, an increase in the twisting of the double helicene core upon charging was observed. The negative charge localization at the central core has been identified by theoretical calculations, which are in full agreement with X‐ray crystallographic and NMR spectroscopic results. Notably, it was confirmed that the two‐electron reduction of OBO‐fused double[5]helicene is reversible.

## Graphical abstract

**Adding extra twist**: An increase in the twisting of the double helicene core upon addition of two electrons has been revealed in the crystallographically characterized solvent‐separated ion triplet (SSIT) and contact‐ion pair (CIP) products. The negative charge localization at the central core has been identified by theoretical calculations and supported by NMR spectroscopic results.

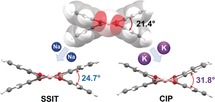

Polycyclic aromatic hydrocarbons (PAHs) have attracted enormous attention due to their intriguing optical and electronic properties.^1^ The possibility to vary the fusion modes of aromatic rings has furnished a great variety of PAH structures. In particular, helicenes, which consist of *ortho*‐fused aromatic rings, are a unique class of compounds with nonplanarity, structural flexibility, and inherent chirality.^2^ Recently, multiple helicenes^3^ with two or more helicene moieties fused together in one PAH framework have gained increasing interest owing to their contorted three‐dimensional structures and multiple chiral states, as well as their applications in organic electronic devices.^4^ Charging PAHs with electrons and elucidating the structural, electronic and supramolecular consequences are of fundamental significance in PAH chemistry.^5^ Particular attention has been paid to nonplanar PAHs,^6^ such as bowl‐shaped corannulene, which was shown to exhibit unique self‐assembly pathways with multiple alkali metal ions upon multi‐electron acquisition.^7^ This has prompted broad explorations of stepwise reduction reactions of nanocarbon systems with different framework topologies.^8^ However, although mobility of alkali metals in helicenes has been recently probed theoretically,^9^ chemical reduction of the emerging multiple helicenes and the resulting structural changes have never been investigated.

On the other hand, incorporation of heteroatoms into PAHs has been an effective strategy to modulate their physicochemical properties.^10^ Among various heteroatoms, boron (B) is particularly appealing because of its Lewis acidity and electron‐accepting character, but its intrinsic instability towards oxygen and moisture poses challenges for the synthesis of B‐doped PAHs.^11^ As a result, B‐fused helicenes are still very rare and they are mostly stabilized by adjacent nitrogen (N) or oxygen (O) atoms.^12^ The concomitant incorporation of B and N/O atoms enhances the stability, but compromises the electron‐accepting properties. To our knowledge, the electron‐accepting behavior of B‐fused helicenes or multiple helicenes has never been revealed. Recently, we have developed a new type of OBO‐fused double helicenes with excellent stability.12d, 12e, 12j Herein, we disclose the chemical reduction of an OBO‐fused double [5]helicene (**1**)12e, 12f with sodium (Na) and potassium (K) metals (Scheme 1), revealing enhanced structural distortions and counterion‐dependent solid‐state structures. This work represents the first study on the effects of adding electrons to double helicenes and demonstrates that the OBO unit can still serve as an electron‐accepting site in PAHs even in the presence of O‐atoms.

**Scheme 1:**
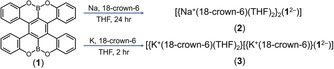
Chemical reduction of OBO‐fused double [5]helicene **1** with Na and K metals to afford the reduced products **2** and **3**.

The OBO‐fused double [5]helicene **1** was synthesized according to the reported procedure.12e The chemical reduction of **1** with Na and K metals in THF proceeds through two distinctive steps characterized by an intermediate red‐brown color followed by a persistent blue‐green color. Since no further changes have been observed even upon prolonged reaction time (Figures S1, S2, S4, S5, Supporting Information), the UV/Vis data pointed out the formation of a stable doubly‐reduced state of **1**. The resulting products have been successfully crystallized as sodium and potassium salts (Scheme 1) from THF solutions in the presence of 18‐crown‐6 ether, which facilitated crystallization. The products were isolated as bulk single‐crystalline materials and fully characterized. The X‐ray diffraction analysis confirmed the formation of a solvent‐separated ion triplet (SSIT) with sodium ions, [{Na^+^(18‐crown‐6)(THF)_2_}_2_(**1** ^2−^)] (**2**), which is crystallized with two interstitial THF molecules as **2**⋅2 THF. The second product gives rise to a contact‐ion pair (CIP) with one bound potassium cation, [{K^+^(18‐crown‐6)(THF)_2_}[{K^+^(18‐crown‐6)}(**1** ^2−^)]] (**3**), which is crystallized with a one/half interstitial THF molecule as **3**⋅0.5 THF (See Supporting Information for more details).^13^

The UV/Vis spectra of the doubly‐reduced products are characterized by the appearance of two new absorption peaks with *λ*_max_ at 541 and 753 nm in **2**, 535 and 773 nm in **3** (Figures S3, S6). The anions **1** ^2−^ are stable in THF solution and exhibit characteristic ^1^H NMR spectra at 25 °C (Figure 1). The addition of two electrons to **1** is accompanied by high‐field shifts of the aromatic proton signals in **2** and **3**. This change can be attributed to the increased electronegativity of the double helicene core, including the peripheral six‐membered rings, upon two‐electron charging. Interestingly, the observed shifts are the largest at the position *d* (ca. 4.1 ppm) in contrast to those for *a*, *b* and *c* (ca. 1.5, 1.8 and 0.8 ppm, respectively). A direct comparison of ^1^H NMR data for **2** and **3** shows that aromatic protons of **1** ^2−^ in the latter are slightly more deshielded, most probably as a result of direct metal binding existing in solution. As the temperature goes down to −80 °C (Figure S16), an additional slight down‐field shift is observed in **3**, which is again indicative of the persisting interaction between **1** ^2−^ and {K^+^(18‐crown‐6)} cations in solution.

**Figure 1:**
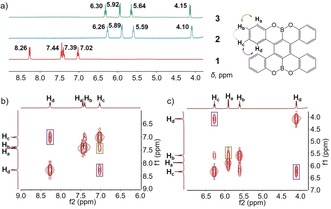
a) ^1^H NMR spectra of **1**, **2** and **3**, b) ^1^H‐^1^H COSY spectrum of **1**, c) ^1^H‐^1^H COSY spectrum of **2**, in THF‐*d_8_* at 25 °C, aromatic region.

The large difference in chemical shifts of aromatic protons upon reduction prompted us to gain deeper insights into the charge distribution of the dianionic species **1** ^2−^ by conducting density functional theory (DFT) calculations. Electrostatic potential (ESP) maps of the parent **1** and its dianion **1** ^2−^ were calculated at the B3LYP/6‐311++G(d,p) level for comparison. In the neutral state (**1**), there is only a small charge difference over the whole molecule (Figure 2). In contrast, in the doubly‐reduced state (**1** ^2−^), a significant localization of the negative charge is observed in the central benzene ring, to which the two B atoms are connected. This charge distribution pattern may help to identify the potential metal binding site and also to explain the significant effect of the two‐electron acquisition process on the H_d_ protons in the adjacent fjord region, as indicated by the ^1^H NMR data. The observed high‐field shifts of aromatic signals in **2** and **3** pointed toward the reduced aromaticity of **1** ^2−^ and stimulated detailed structural analysis of the products.

**Figure 2:**
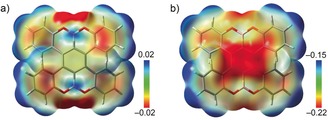
Electrostatic potential (ESP) maps of a) neutral **1** and b) its dianionic congener **1** ^2−^.

In the crystal structure of **2**, both [Na^+^(18‐crown‐6)(THF)_2_] cations are solvent‐separated from the **1** ^2−^ core (Figure 3), allowing for the structural analysis of the “naked” OBO‐fused double [5]helicene dianion. The Na^+^ ions are axially coordinated by one 18‐crown‐6 ether molecule (Na⋅⋅⋅O_crown_, 2.635(5)–2.821(5) Å) and capped by two THF molecules (Na⋅⋅⋅O_THF_, 2.270(16)–2.292(13) Å) with all Na⋅⋅⋅O distances being close to those previously reported.^14^ The average B−C bond length distance in **2** of 1.518(7) Å is very similar to that in **1** (1.514(6) Å),12e consistent with its single bond character (Table S3). The average B−O bond length distances in **1** and **2** (1.377(5) and 1.371(8) Å, respectively) also correspond to the typical values for the three‐coordinated boron‐oxygen compounds.^15^

**Figure 3:**
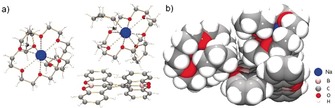
Molecular structure of **2**, a) ball‐and‐stick model, b) space‐filling model.

Analysis of the crystal structure of **3** (Figure 4) revealed that one K^+^ ion is bound to the central six‐membered ring of **1** ^2−^ in an *η* ^4^‐mode, with the corresponding K⋅⋅⋅C bond distances ranging from 3.108(3) to 3.360(3) Å (Figure 4 c). This binding mode is in good agreement with the negative charge localization pattern observed in the ESP map analysis of **1** ^2−^ (Figure 2 b). The coordination of this K^+^ ion is completed by one 18‐crown‐6 ether molecule with the K⋅⋅⋅O_crown_ bond length distances ranging from 2.769(2) to 3.004(2) Å. The second [K^+^(18‐crown‐6)(THF)_2_] cation is solvent‐separated from the anionic [{K^+^(18‐crown‐6)}(**1** ^2−^)]^−^ complex. This K^+^ ion is fully wrapped by one 18‐crown‐6 ether (K⋅⋅⋅O_crown_, 2.756(5)–2.843(5) Å) and two THF molecules (K⋅⋅⋅O_THF_, 2.786(3) Å and 2.848(3) Å) with all K⋅⋅⋅O bond length distances being in the range of previously reported.12j, 16

**Figure 4:**
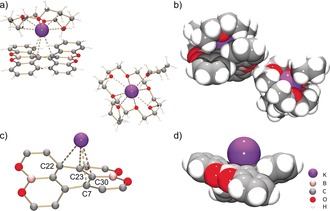
Molecular structure of **3**, a) ball‐and stick model, b) space‐filling model, c) metal coordination in ball‐and‐stick model (fragment of **1** ^2−^ is shown for clarity; K−C7: 3.108(3) Å, K−C22: 3.272(3) Å, K−C23: 3.360(3) Å, K−C30: 3.151(3) Å), d) metal coordination in space‐filling model.

Analysis of C−C bonds of **1** ^2−^ in **2** and **3** vs. neutral **1** (Table S2) reveals that the main changes are associated with the central part of the helicene core (Figure 5 a). Specifically, the bond length distances of C6−C7, C8−C9, C22−C21 and C23−C24 are shortened in both **2** and **3**, while the adjacent C7−C8 and C22−C23 bonds become notably elongated. These structural changes are fully consistent with the decreased aromaticity of **1** ^2−^. As shown above, the significant localization of negative charge has been detected at the central benzene site of **1** ^2−^ and that can explain the observed bond distance alterations.

**Figure 5:**
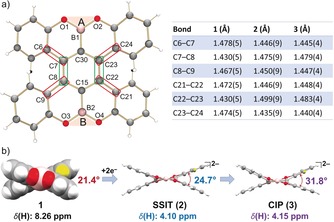
a) The helicene core in **1**, **2**, and **3** along with the table of selected C−C distances. The bond is circled in red/green when the distance is shorter/longer in **2** and **3** compared to that in **1**, b) Twisting of the helicene core upon reduction (**2** and **3**).

The key structural feature of helicenes is the twist of the aromatic core. In order to evaluate the possible changes upon two‐electron addition, the selected torsion and dihedral angles of **1** ^2−^ in **2** and **3** were measured and compared with those in **1**. The torsion angles for C6‐C7‐C8‐C9 and C21‐C22‐C23‐C24 in **2** are 34.2° and 35.1°, respectively. Notably, both are larger than those in **1** (32.3° and 28.2°, Table S4), illustrating an increase in the core twisting upon two‐electron acquisition. The corresponding angles for **1** ^2−^ are further increased in **3** (34.3° and 35.8°, respectively), showing the clear influence of metal binding. The measured A/B plane angle (Figure 5 a) of 24.7° in **2** also illustrates an increased twist upon charging in comparison with that in **1** (21.4°). Moreover, the presence of metal binding renders the dianion core in **3** even more twisted with the A/B plane angle increased to 31.8° (Figure 5 b). These findings are remarkable since efficient charge delocalization in non‐helical PAHs is expected to be favored by a planarized core.

The solid state structures of **2** and **3** (Figure 6) differ from the crystal structure of parent **1** that is based on intermolecular π‐π interactions.12e Analysis of packing in both structures identifies only weak intermolecular C−H⋅⋅⋅π contacts between the **1** ^2−^ anions and adjacent 18‐crown‐6 ether cationic moieties, with the shortest distances ranging from 2.551(8) Å to 2.628(8) Å in **2** and those of 2.607(4) Å in **3**.

**Figure 6:**
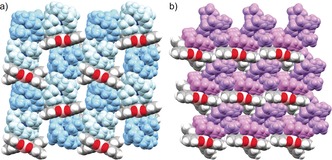
a) Solid state packing in **2** and b) Solid state packing in **3**, space‐filling models. The [Na^+^(18‐crown‐6)(THF)_2_] moieties are shown in different shades of blue. The [K^+^(18‐crown‐6)(THF)_2_] and [K^+^(18‐crown‐6)] moieties are shown in different shades of purple.

Finally, it was shown that the doubly‐reduced **1** ^2−^ helicene can be reversibly re‐oxidized back to the neutral state, as confirmed by ^1^H NMR and DART‐MS spectroscopy (Figures S18, S19). This indicates the inherent flexibility and stability of the OBO‐fused double [5]helicene core towards reduction/oxidation cycles.

In summary, the first chemical reduction study of a double helicene and successful X‐ray structural characterization of the reduced products of the OBO‐fused double [5]helicene (**1**) has been accomplished. The use of two different alkali metals, Na and K, has allowed to switch metal binding on and off in the counterion‐dependent crystal structures. The direct comparison of the “naked” and complexed forms of the dianion **1** ^2−^ reveals a structural distortion of the helicene core upon two‐electron acquisition that is further enhanced by metal binding. Analysis of the charge density distribution using DFT methods has demonstrated that the OBO unit can serve as an electron‐accepting site even in the presence of O‐atoms. Notably, the observed changes in electronic structure of **1** ^2−^ are accompanied by significant shifts of aromatic proton signals, as detected by ^1^H NMR spectroscopy. The reduced aromaticity of the **1** ^2−^ core is clearly manifested by the CC bond length alterations and de‐shielding of the aromatic protons in the ^1^H NMR spectra. Importantly, the doubly‐reduced helicene **1** ^2−^ can be reversibly re‐oxidized back to the neutral state, suggesting potential applications for redox‐driven chiroptical switches.

## Conflict of interest

The authors declare no conflict of interest.

## Supporting information

As a service to our authors and readers, this journal provides supporting information supplied by the authors. Such materials are peer reviewed and may be re‐organized for online delivery, but are not copy‐edited or typeset. Technical support issues arising from supporting information (other than missing files) should be addressed to the authors.

Supplementary
