# Supplementary material for: Charging OBO‐Fused Double [5]Helicene with Electrons
Source: Angew Chem Int Ed Engl. 2019 Sep 9;58(42):14969–73. doi: 10.1002/anie.201908658 (PMC6916263; doi:10.1002/anie.201908658)
Supplement: Supplementary file 1 — Supplementary [file ANIE-58-14969-s001.pdf]

Angewandte Chemie
Eine Zeitschrift der Gesellschaft Deutscher Chemiker

Supporting Information

Charging OBO-Fused Double [5]Helicene with Electrons

Zheng Zhou, Xiao-Ye Wang, Zheng Wei, Klaus Müllen, and Marina A. Petrukhina**

anie_201908658_sm_miscellaneous_information.pdf

Supporting Information

I. Materials and Methods S3

Preparation of [{Na^+(18-crown-6)$(THF)_2$}$_2$(1^{2-})]·2THF (2·2THF) S3

Preparation of [{K^+(18-crown-6)$(THF)_2$}{K^+(18-crown-6)}(1^{2-})]·0.5THF (3·0.5THF) S3

II. UV/Vis Spectroscopy Investigation S4

Probe preparation of Na/1 in THF S4

Figure S1. UV/Vis spectra of Na/**1** in THF S4

Probe preparation of Na/18-crown-6/1 in THF S5

Figure S2. UV/Vis spectra of Na/18-crown-6/**1** S5

Figure S3. UV/Vis spectra of *in situ* generated $[Na^+(THF)_n]_2[\mathbf{1}^{2-}]$ and $[Na^+$(18-crown-6)$(THF)_n]_2[\mathbf{1}^{2-}]$ *vs.* dissolved crystals of **2** S5

Probe preparation of K/1 in THF S6

Figure S4. UV/Vis spectra of K/**1** in THF S6

Probe preparation of K/18-crown-6/1 in THF S6

Figure S5. UV/Vis spectra of K/18-crown-6/**1** S7

Figure S6. UV/Vis spectra of *in situ* generated $[K^+(THF)_n]_2[\mathbf{1}^{2-}]$ and $[K^+$(18-crown-6)$(THF)_n]_2[\mathbf{1}^{2-}]$ *vs*. dissolved crystals of **3** S7

III. ^{1}H NMR Spectroscopy Investigation S8

Probe preparation of Na/18-crown-6/1 in THF-*d*$_8$ S8

Figure S7. Variable-temperature ^{1}H NMR spectra of *in situ* generated $[Na^+$(18-crown-6)$(THF)_n]_2[\mathbf{1}^{2-}]$, aromatic region S8

Figure S8. ^{1}H NMR spectra of *in situ* generated $[Na^+$(18-crown-6)$(THF)_n]_2[\mathbf{1}^{2-}]$ at 25 °C and –80 °C S9

Figure S9. ^{1}H NMR spectra of *in situ* generated $[Na^+$(18-crown-6)$(THF)_n]_2[\mathbf{1}^{2-}]$ at 25 °C and –80 °C with integrations, aromatic region S9

Probe preparation of 2 in THF-*d*$_8$ S10

Figure S10. Variable-temperature ^{1}H NMR spectra of **2** in THF-*d*$_8$, aromatic region S10

Figure S11. ^{1}H NMR spectra of **2** at 25 °C and –80 °C S11

Figure S12. ^{1}H NMR spectra of **2** at 25 °C and –80 °C with integrations, aromatic region S11

Probe preparation of K/18-crown-6/1 in THF-*d*$_8$ S12

Figure S13. Variable-temperature ^{1}H NMR spectra of *in situ* generated $[K^+$(18-crown-6)$(THF)_n]_2[\mathbf{1}^{2-}]$, aromatic region S12

Figure S14. ^{1}H NMR spectra of *in situ* generated $[K^+$(18-crown-6)$(THF)_n]_2[\mathbf{1}^{2-}]$ at 25 °C and –80 °C S13

Figure S15. ^{1}H NMR spectra of *in situ* generated $[K^+$(18-crown-6)$(THF)_n]_2[\mathbf{1}^{2-}]$ at 25 °C and –80 °C with integrations, aromatic region S13

Probe preparation of 3 in THF-*d*$_8$ S14

Figure S16. Variable-temperature ^{1}H NMR spectra of **3**, aromatic region S14

Figure S17. ^{1}H NMR spectra of **3** at 25 °C and –80 °C S14
Figure S18. ^{1}H NMR spectra of **3** at 25 °C and –80 °C with integrations, aromatic region S15
IV. Redox Reversibility Study S16
NMR Probe Preparation S16
Figure S19. ^{1}H NMR spectra of **1**, *in situ* generated $\mathbf{1}^{2-}$, and its quenched product S16
DART-MS Sample Preparation S17
Figure S20. DART-MS spectra of **1** and the quenched product of *in situ* generated $\mathbf{1}^{2-}$ S17
V. Crystal Structure Solution and Refinement Details S18
Table S1. Crystal Data and Structure Refinement Parameters for **2** and **3** S19
Figure S21. ORTEP drawing of **2** S20
Figure S22. ORTEP drawing of **3** S20
Table S2. Selected C–C bond distances in **1**, **2** and **3** S21
Table S3. Selected bond distances in **1**, **2** and **3** S22
Table S4. Selected torsion angles and plane angles in **1**, **2** and **3**, along with labeling scheme S22
VI. Theoretical Calculations S23
Table S5. Cartesian coordinates of the neutral **1** S23
Table S6. Cartesian coordinates of the dianion $\mathbf{1}^{2-}$ S24
VII. References S26

I. Materials and Methods

All manipulations were carried out using break-and-seal[1] and glove-box techniques under an atmosphere of argon. Tetrahydrofuran (THF) and hexanes were purchased from Pharmco-Aaper and were dried over Na/benzophenone and distilled prior to use. THF-d_8 was purchased from Sigma Aldrich, dried over NaK_2 alloy and vacuum-transferred. Sodium, potassium and 18-Crown-6 ether were purchased from Sigma Aldrich and used as received. OBO-double[5]helicene ($C_{30}H_{16}B_2O_4$, **1**) was prepared according to the previously reported procedure[2] and purified by sublimation. The UV/Vis spectra were recorded on a PerkinElmer Lambda 35 spectrometer. The ^{1}H NMR spectra were measured using Bruker AC-400 and Ascend-500 spectrometers and referenced to the resonances of the solvent used.

Preparation of [{Na^+(18-crown-6)$(THF)_2$}$_2$($1^{2–}$)]·2THF (2·2THF)

THF (1.2 mL) was added to a flask containing excess Na (1.1 mg, 8 eq.), 18-crown-6 ether (5.7 mg, 0.022 mmol) and **1** (5 mg, 0.011 mmol). The initial color of the mixture was pale yellow (neutral ligand). Within 15 minutes, the color turned to dark red-brown and then to dark green-blue (in *ca*. 60 min). After a total of 24 h of stirring, the resulting suspension was filtered; the dark green-blue filtrate was layered with hexanes (0.9 mL) and placed at 5 °C. Dark green-blue blocks were present in high yield after 4 days. Yield: 85%. UV/Vis (THF): λ_{max} 313, 398, 541 and 753 nm. ^{1}H NMR (THF-d_8, –80 °C): δ = 1.78 (16H, THF), 3.54 (48H, 18-crown-6), 3.61 (16H, THF), 4.00 (4H, $\mathbf{1}^{2–}$), 5.59 (4H, $\mathbf{1}^{2–}$), 5.88 (4H, $\mathbf{1}^{2–}$), 6.26 (4H, $\mathbf{1}^{2–}$).

Preparation of [{K^+(18-crown-6)$(THF)_2$}{K^+(18-crown-6)}($1^{2–}$)]·0.5THF (3·0.5THF)

THF (1.5 mL) was added to a flask containing excess K (1.1 mg, 6 eq.), 18-crown-6 ether (2.3 mg, 0.009 mmol) and **1** (2 mg, 0.004 mmol). The initial color of the mixture was pale yellow (neutral ligand). Within a few minutes, the color turned to dark red-brown and then to dark green-blue (in *ca*. 50 min). After a total of 2 h of stirring, the resulting suspension was filtered; the dark green-blue filtrate was layered with hexanes (1.2 mL) and placed at 5 °C. Dark green-blue blocks were present in good yield after 3 days. Yield: 75%. UV/Vis (THF): λ_{max} 313, 381, 535 and 773 nm. ^{1}H NMR (THF-d_8, –80 °C): δ = 1.78 (16H, THF), 3.54 (48H, 18-crown-6), 3.61 (16H, THF), 4.01 (4H, $\mathbf{1}^{2–}$), 5.60 (4H, $\mathbf{1}^{2–}$), 5.88 (4H, $\mathbf{1}^{2–}$), 6.30 (4H, $\mathbf{1}^{2–}$).

II. UV/Vis Spectroscopy Investigation

Probe preparation of Na/1 in THF:

THF (4 mL) was added to a probe containing excess Na metal (~5 eq.) and **1** (0.02 mg, 4.3×10^{-5} mmol) and UV/Vis spectra were monitored at different reaction times (total 24h) at room temperature.

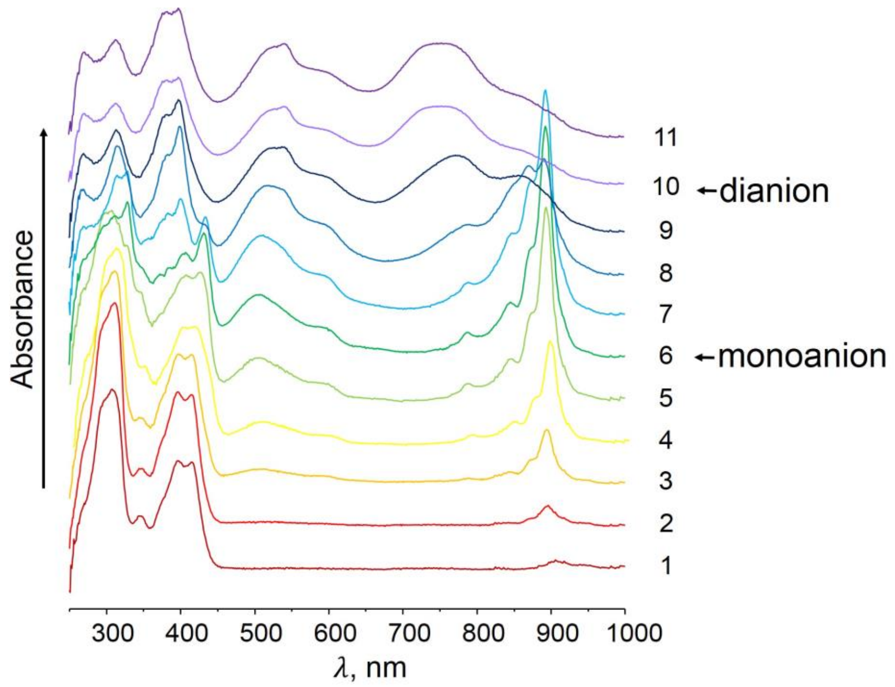

Label	Time (min)
1	1
2	3
3	8
4	15
5	25
6	35
7	45
8	55
9	90
10	120
11	1440

Figure S1. UV/Vis spectra of Na/**1** in THF.

Probe preparation of Na/18-crown-6/1 in THF:

THF (4 mL) was added to a probe containing excess Na metal (~5 eq.), 18-crown-6 (~2.5 eq.) and **1** (0.02 mg, 4.3×10^{-5} mmol) and UV/Vis spectra were monitored at different reaction times (total 24h) at room temperature.

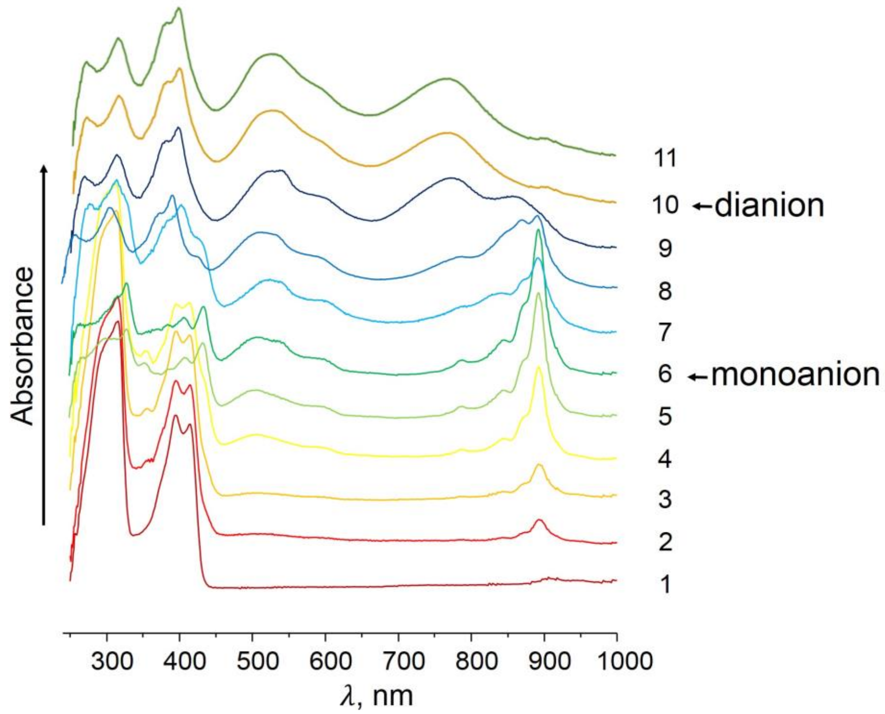

Label	Time (min)
1	1
2	3
3	5
4	15
5	25
6	30
7	40
8	50
9	85
10	120
11	1440

Figure S2. UV/Vis spectra of Na/18-crown-6/**1** in THF.

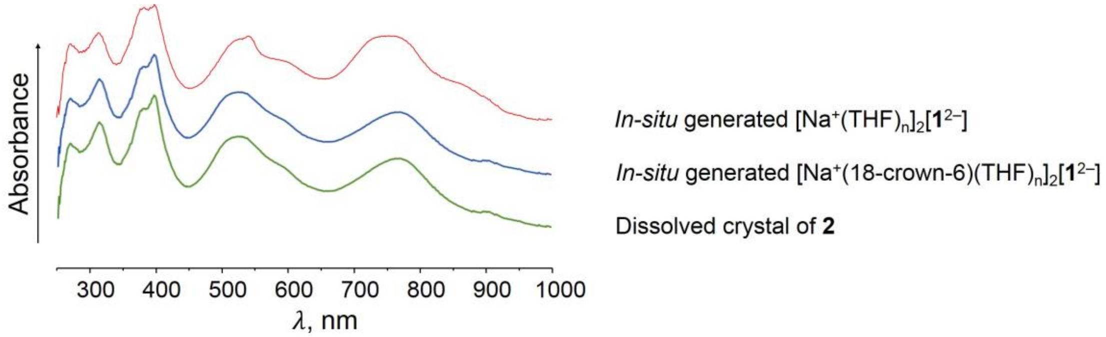

Label	λ_{max} (nm)
Dissolved crystal	313, 398, 541, 753
In-situ with crown	314, 398, 535, 766
In-situ without crown	315, 399, 535, 766

Figure S3. UV/Vis spectra of *in situ* generated $[Na^+(THF)_n]_2[\mathbf{1}^{2-}]$ and $[Na^+(18\text{-crown-}6)(THF)_n]_2[\mathbf{1}^{2-}]$ *vs.* dissolved crystals of **2**·2THF.

Probe preparation of K/1 in THF:

THF (4 mL) was added to a probe containing excess K metal (~4 eq.) and **1** (0.02 mg, 4.3×10^{-5} mmol) and UV/Vis spectra were monitored at different reaction times (total 24h) at room temperature. *Note: after 30 hours the bulk precipitation was observed and that prevented further reaction monitoring.*

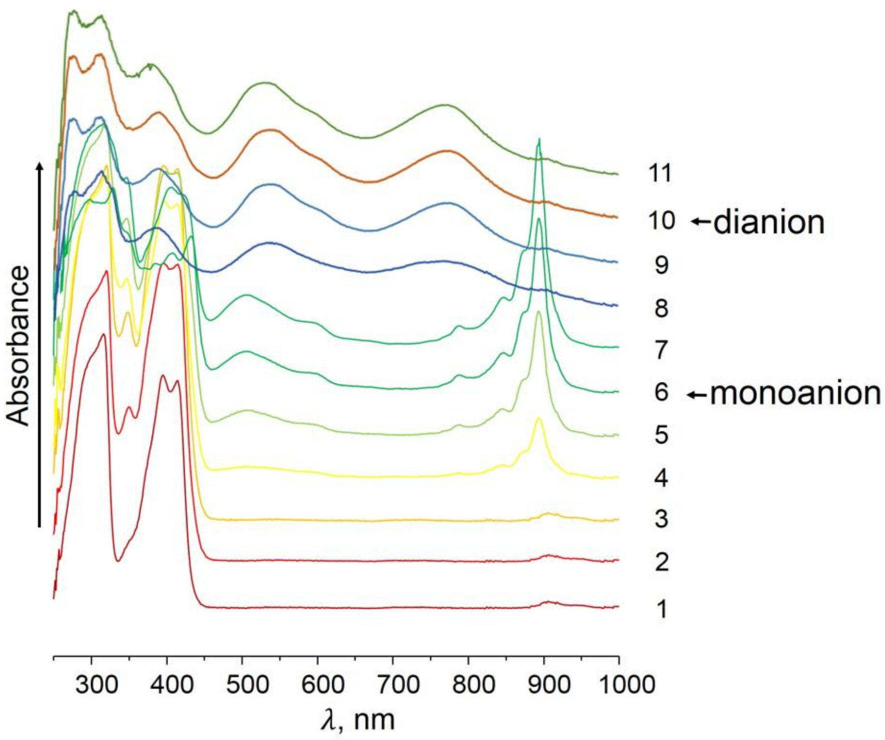

Label	Time (min)
1	1
2	2
3	4
4	10
5	18
6	20
7	25
8	30
9	40
10	50
11	1440

Figure S4. UV/Vis spectra of K/**1** in THF.

Probe preparation of K/18-crown-6/1 in THF:

THF (4 mL) was added to a probe containing excess K metal (~4 eq.), 18-crown-6 (~2.5 eq.) and **1** (0.02 mg, 4.3×10^{-5} mmol) and UV/Vis spectra were monitored at different reaction times (total 24h) at room temperature. *Note: after 30 hours the bulk precipitation was observed and that prevented further reaction monitoring.*

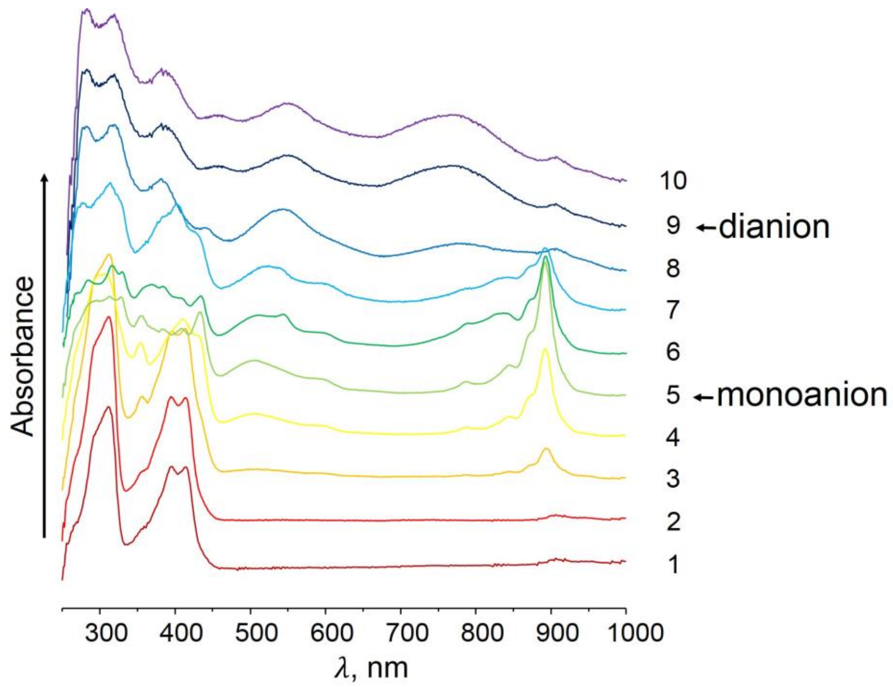

Label	Time (min)
1	1
2	2
3	5
4	10
5	20
6	25
7	30
8	45
9	55
10	1440

Figure S5. UV/Vis spectra of K/18-crown-6/**1** in THF.

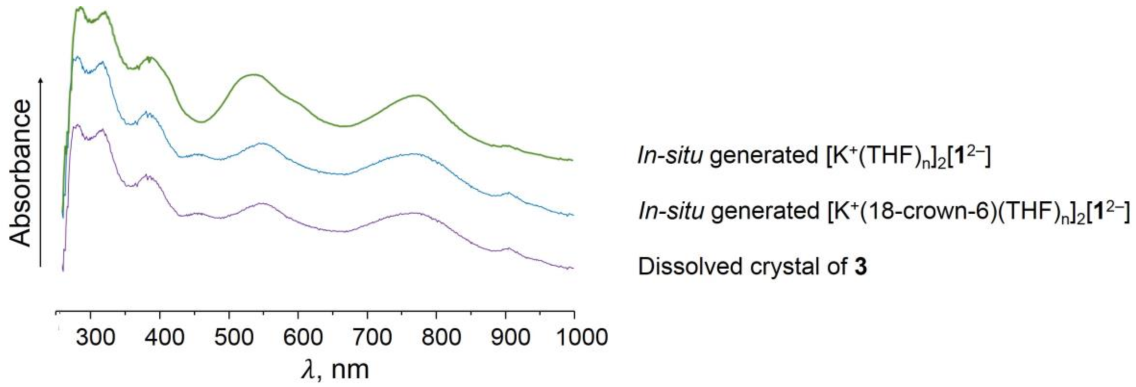

Label	λ_{max} (nm)
Dissolved crystals	313, 381, 535, 773
In-situ with crown	314, 376, 534, 772
In-situ without crown	314, 383, 541, 771

Figure S6. UV/Vis spectra of *in situ* generated $[K^+(THF)_n]_2[\mathbf{1}^{2-}]$ and $[K^+(18\text{-crown-6})(THF)_n]_2[\mathbf{1}^{2-}]$ *vs*. dissolved crystals of **3**.

III. ^{1}H NMR Spectroscopy Investigation

Probe preparation of Na/18-Crown-6/1 in THF-*d*8:

THF-d_8 (0.7 mL) was added to an NMR probe containing excess Na metal (~8 eq.), 18-crown-6 (~2.2 eq.) and **1** (3 mg, 0.007 mmol). The initial color of the mixture was red-brown (monoanion stage). The probe was allowed to sit for 24 hours resulting in a dark green-blue solution (dianion). The Na metal was decanted from the mixture and ^{1}H NMR spectra were monitored.

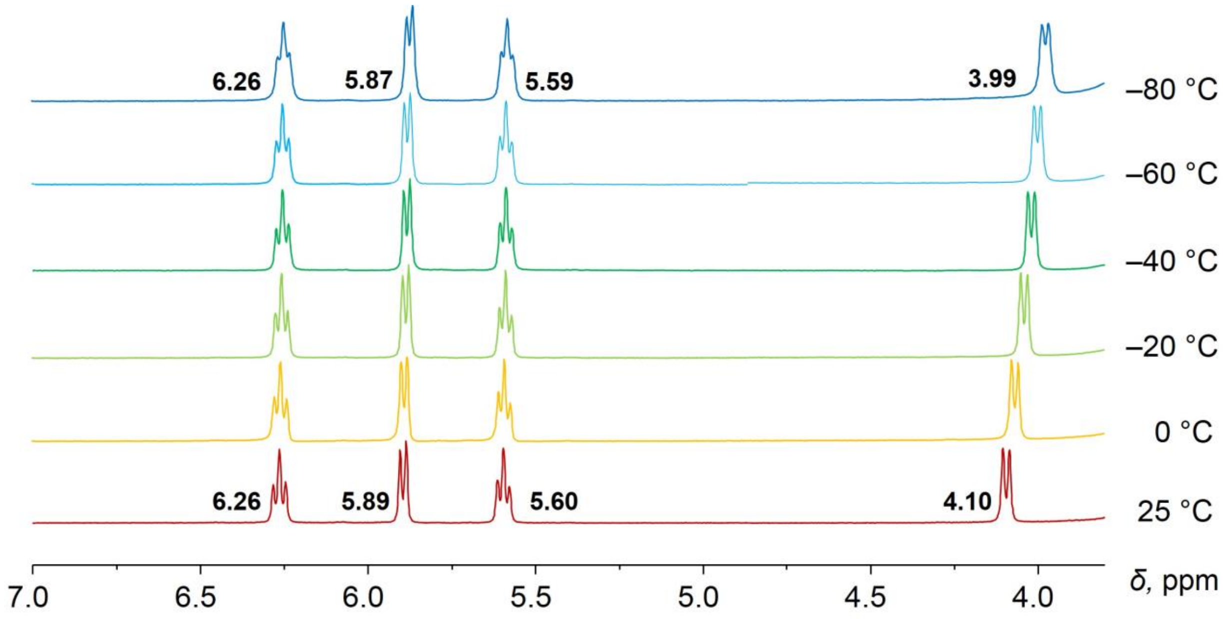

Figure S7. Variable-temperature ^{1}H NMR spectra of *in situ* generated $[Na^{+}(18\text{-crown-}6)(THF)_n]_2[\mathbf{1}^{2-}]$ in THF-d_8, aromatic region.

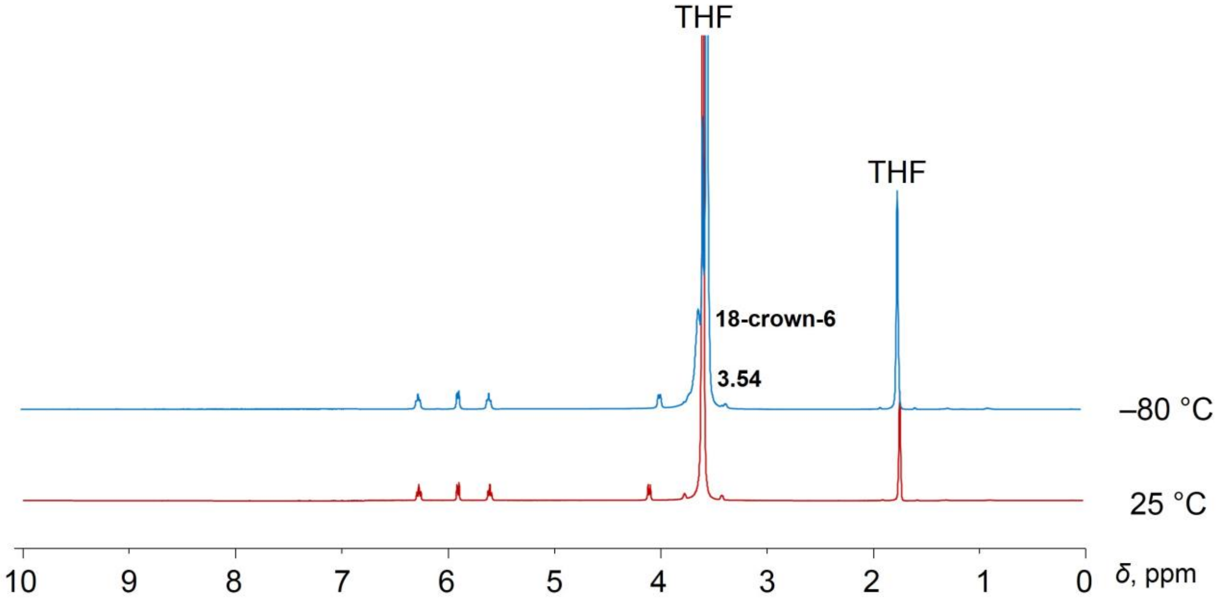

Figure S8. ^{1}H NMR spectra of *in situ* generated $[Na^+(18\text{-crown-6})(THF)_n]_2[\mathbf{1}^{2-}]$ in THF-d_8 at 25 °C and –80 °C.

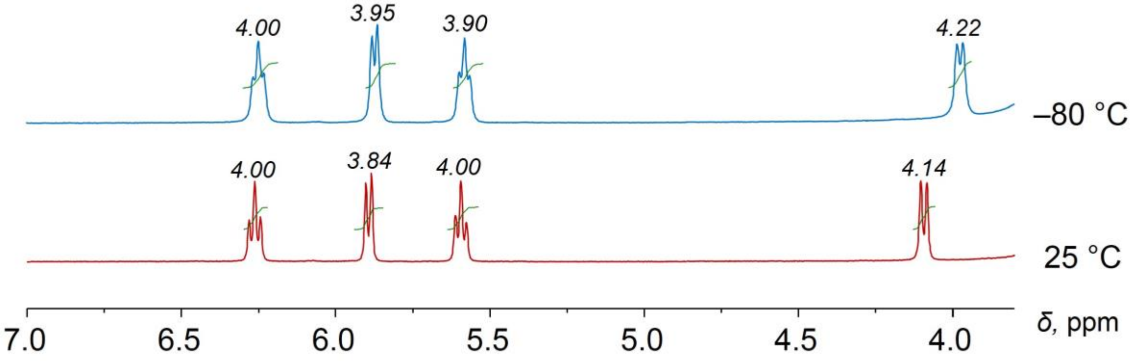

Figure S9. ^{1}H NMR spectra of *in situ* generated $[Na^+(18\text{-crown-6})(THF)_n]_2[\mathbf{1}^{2-}]$ in THF-d_8 at 25 °C and –80 °C with integrations, aromatic region.

Probe preparation of 2 in THF-d_8:

5 mg of crystalline **2**·2THF were washed several times with hexanes and dried *in-vacuo*. THF-d_8 (0.7 mL) was added to the crystals and the solution was transferred to an NMR tube.

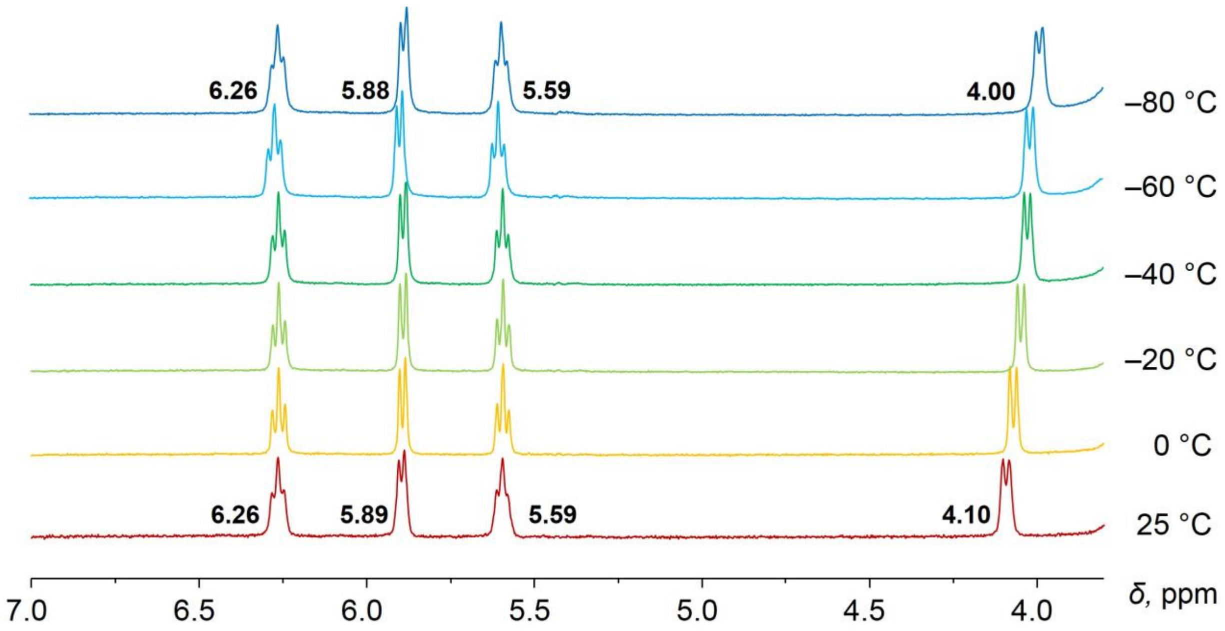

Figure S10. Variable-temperature ^{1}H NMR spectra of **2** in THF-d_8, aromatic region.

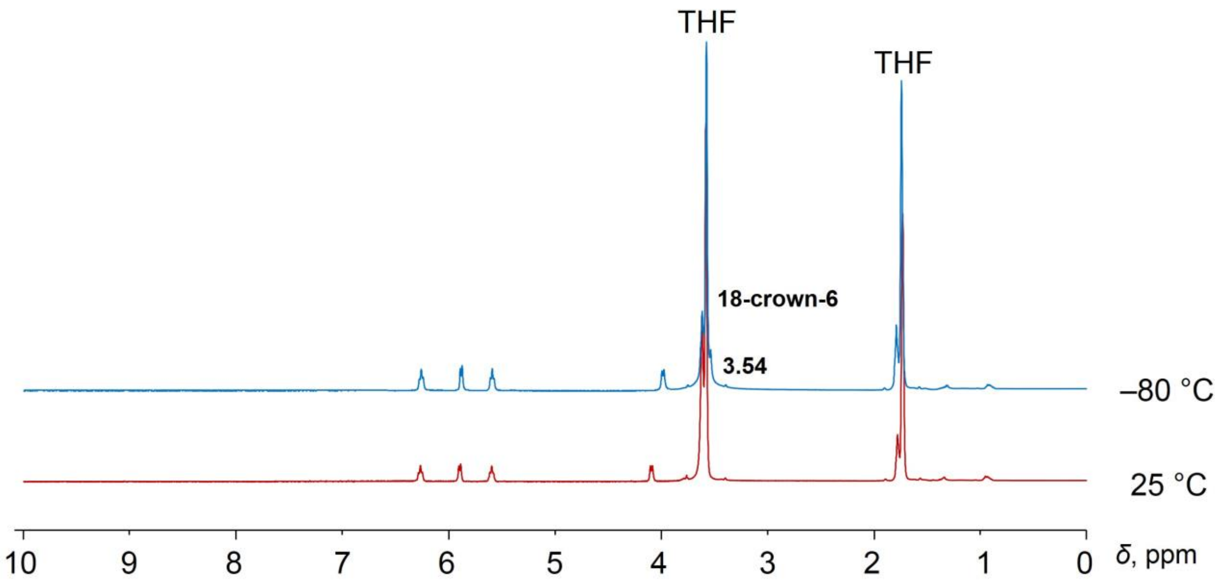

Figure S11. ^{1}H NMR spectra of **2** in THF-d_8 at 25 °C and –80 °C.

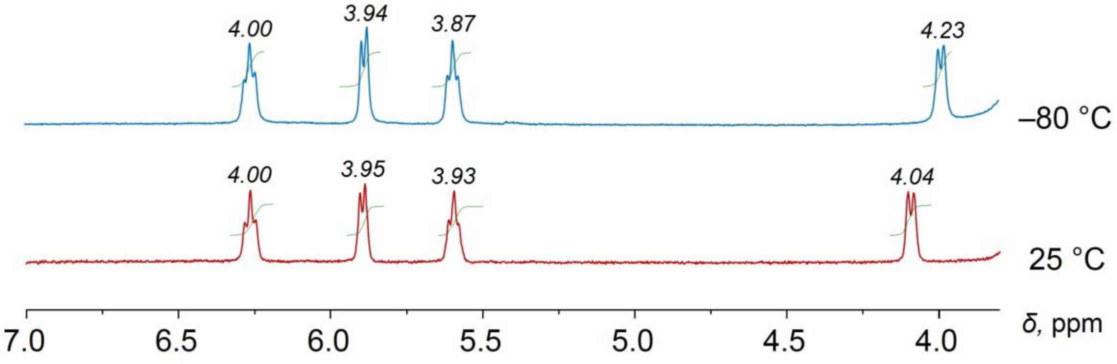

Figure S12. ^{1}H NMR spectra of **2** in THF-d_8 at 25 °C and –80 °C with integrations, aromatic region.

Probe preparation of K/18-crown-6/1 in THF-d_8:

THF-d_8 (0.7 mL) was added to an NMR tube containing excess K metal (~7 eq.), 18-crown-6 (~2.1 eq.) and **1** (3 mg, 0.0066 mmol). The initial color of the mixture was red-brown. The probe was allowed to sit for 24 hours resulting in a dark blue-green solution. The Na metal was decanted from the mixture and ^{1}H NMR spectra were monitored.

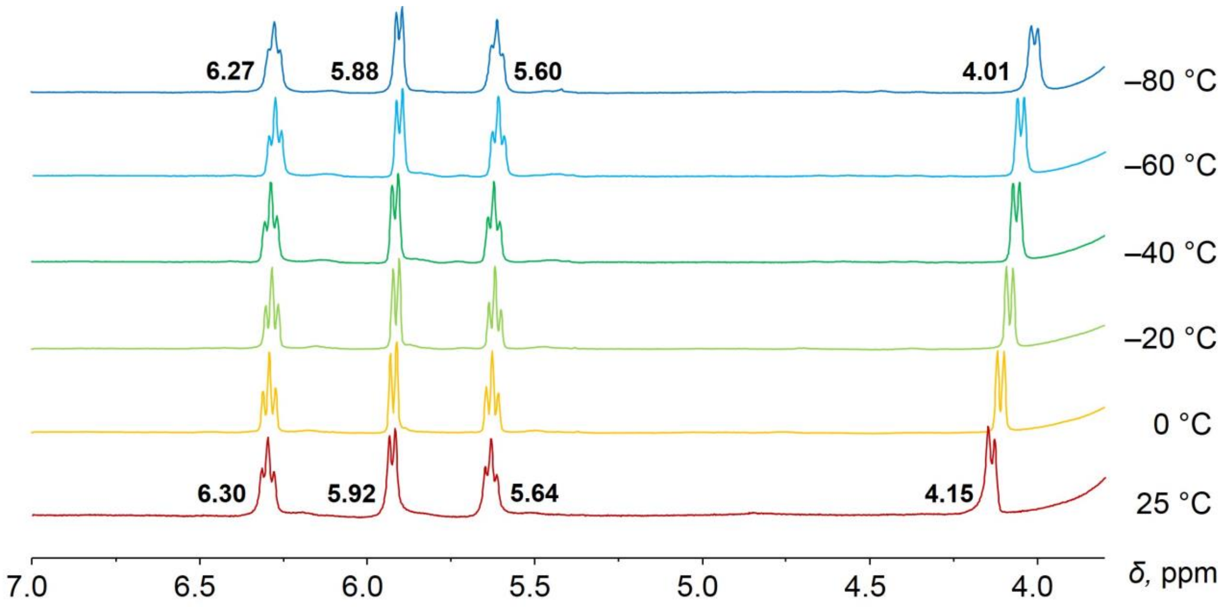

Figure S13. Variable-temperature ^{1}H NMR spectra of *in situ* generated [K^+(18-crown-6)(THF)$_n$]$_2$[$\mathbf{1}^{2-}$] in THF-d_8, aromatic region.

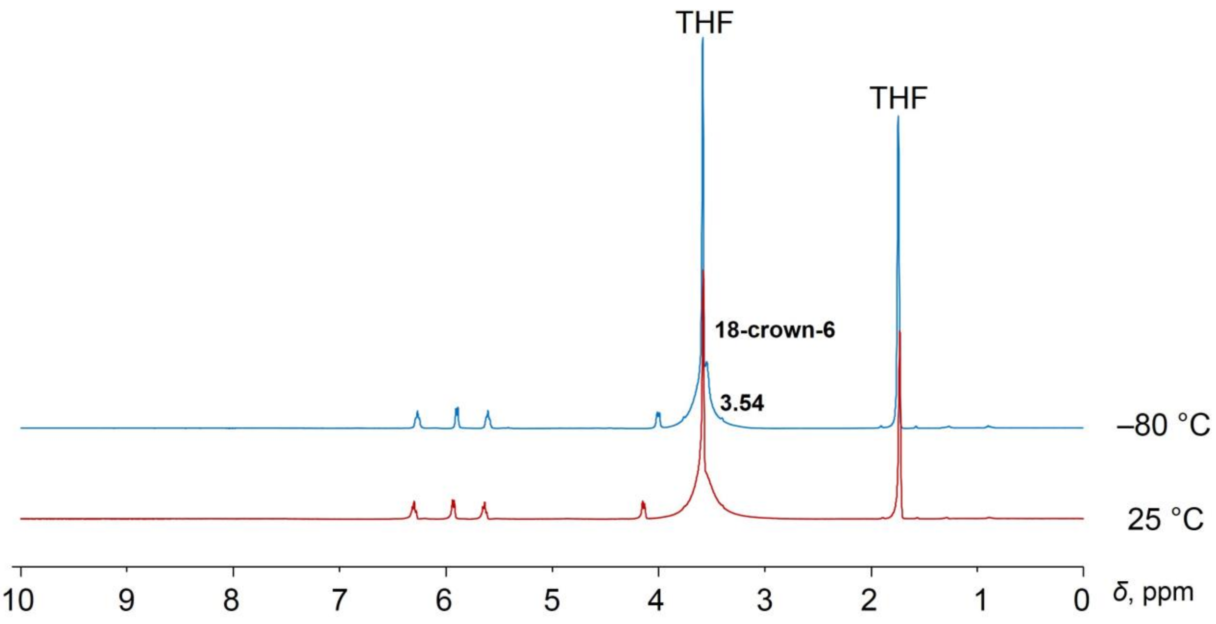

Figure S14. ^{1}H NMR spectra of *in situ* generated $[K^+(18\text{-crown-6})(THF)_n]_2[\mathbf{1}^{2-}]$ in THF-d_8 at 20 °C and –80 °C.

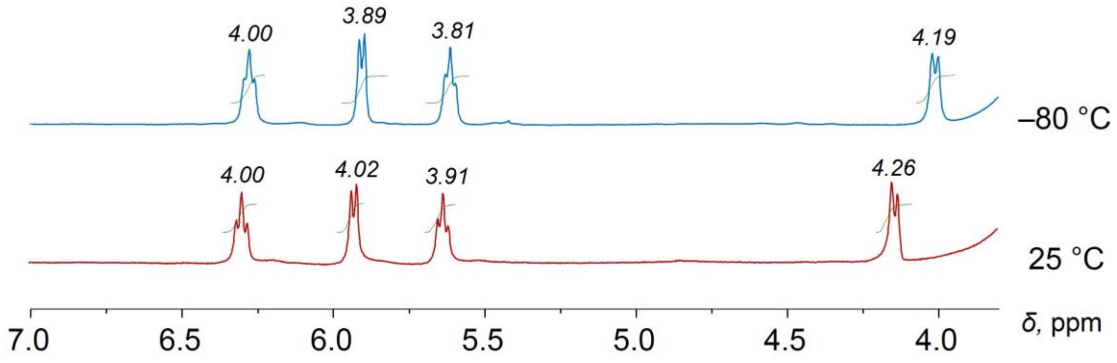

Figure S15. ^{1}H NMR spectra of *in situ* generated $[K^+(18\text{-crown-6})(THF)_n]_2[\mathbf{1}^{2-}]$ in THF-d_8 at 20 °C and –80 °C with integrations, aromatic region.

Probe preparation of 3 in THF-d_8:

5 mg of crystalline **3**·0.5THF were washed several times with hexanes and dried *in-vacuo*. THF-d_8 (0.7 mL) was added to the crystals and the solution was transferred to an NMR tube.

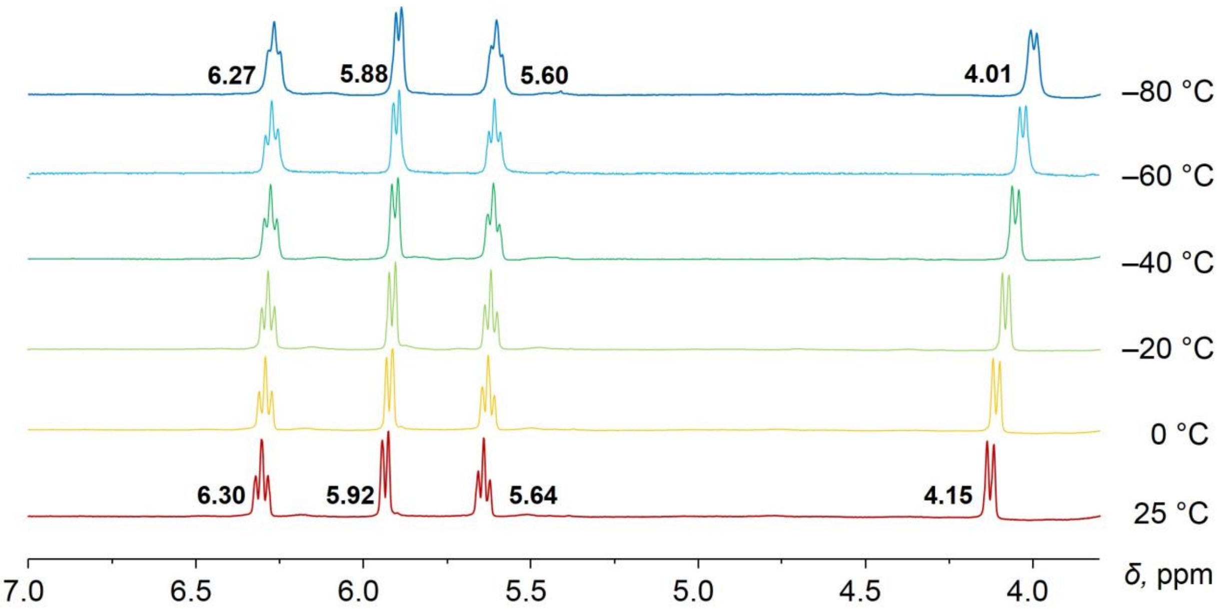

Figure S16. Variable-temperature ^{1}H NMR spectra of **3** in THF-d_8, aromatic region.

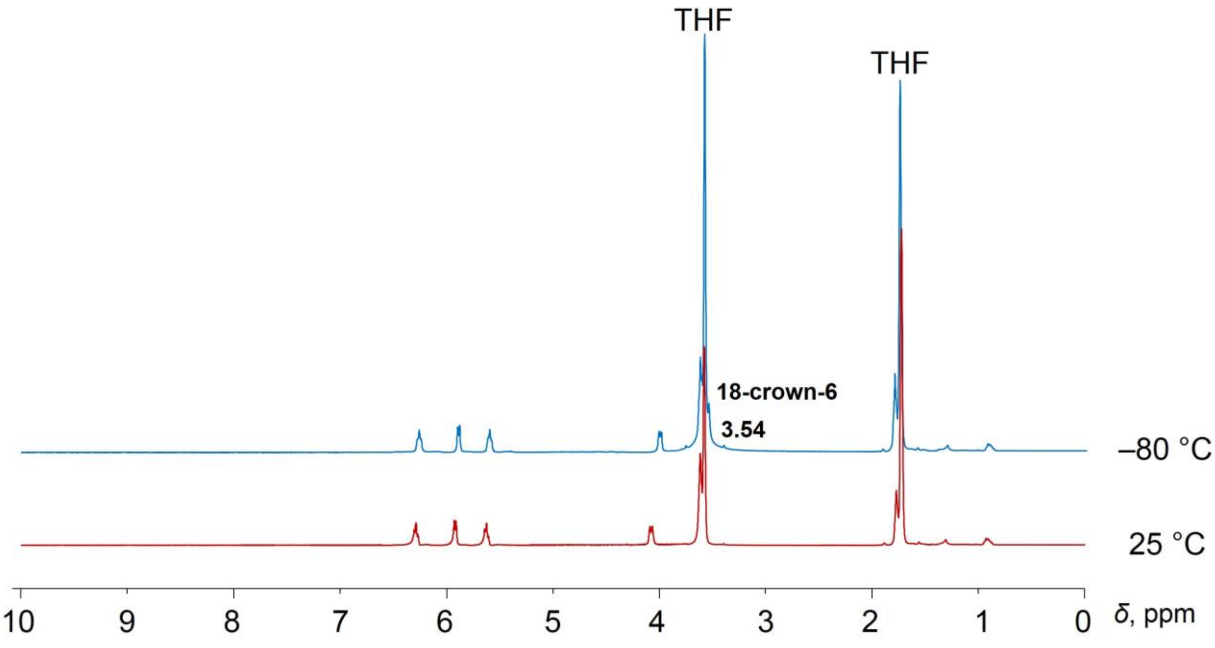

Figure S17. ^{1}H NMR spectra of **3**·in THF-d_8 at 20 °C and –80 °C.

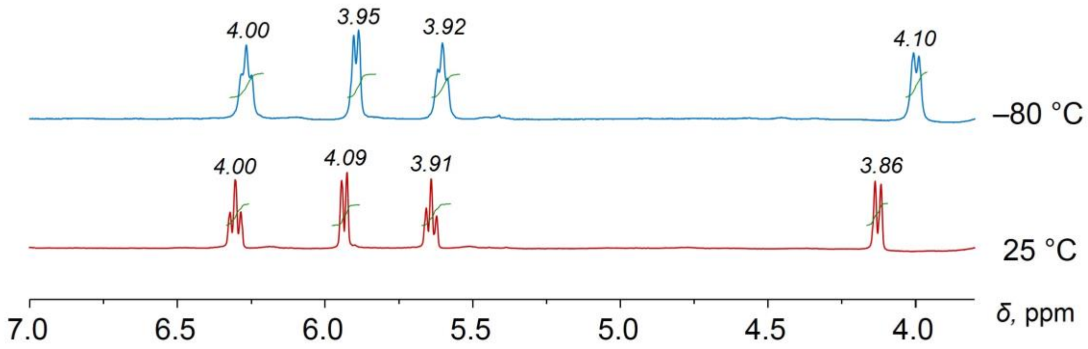

Figure S18. ^{1}H NMR spectra of **3** in THF-d_8 at 25 °C and –80 °C with integrations, aromatic region.

IV. Redox Reversibility Study

NMR Probe Preparation

1 (5 mg, 0.0108 mmol), one piece of Na metal (~10 eq.) and 18-crown-6 ether (5.7 mg, 0.0216 mmol) were added into an NMR tube, followed by addition of 0.7 mL of fresh THF-d_8. The tube was sealed under argon. The ^{1}H NMR spectrum of **1** was collected immediately, and that of $\mathbf{1}^{2-}$ was collected after 24 hours. The solution was then exposed to air by opening the tube, and its spectrum was recorded as "quenched".

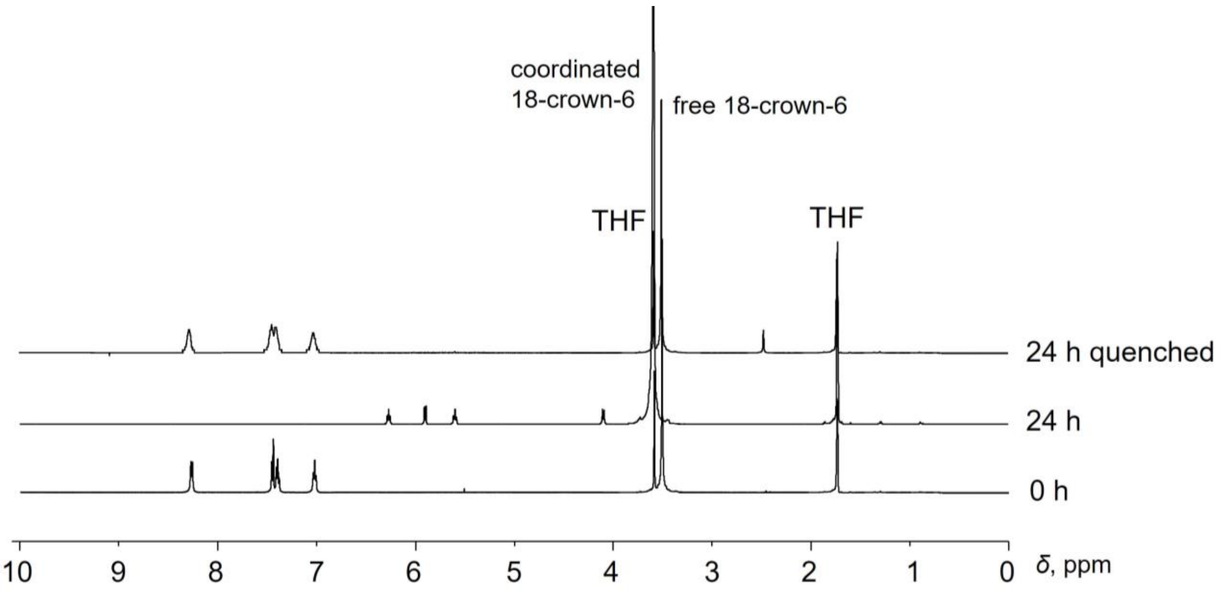

Figure S19. ^{1}H NMR spectra of **1**, *in situ* generated $\mathbf{1}^{2-}$, and its quenched product at 25 °C in THF-d_8.

DART-MS Sample Preparation

2 mg of **1** and a piece of Na metal (excess) were added into a glass tube, followed by the addition of 2.0 mL of fresh THF. The tube was sealed under argon. After the formation of doubly-reduced product of **1** in 48 hours, the solution was exposed to air by opening the tube, and the spectrum was recorded as quenched $\mathbf{1}^{2-}$.

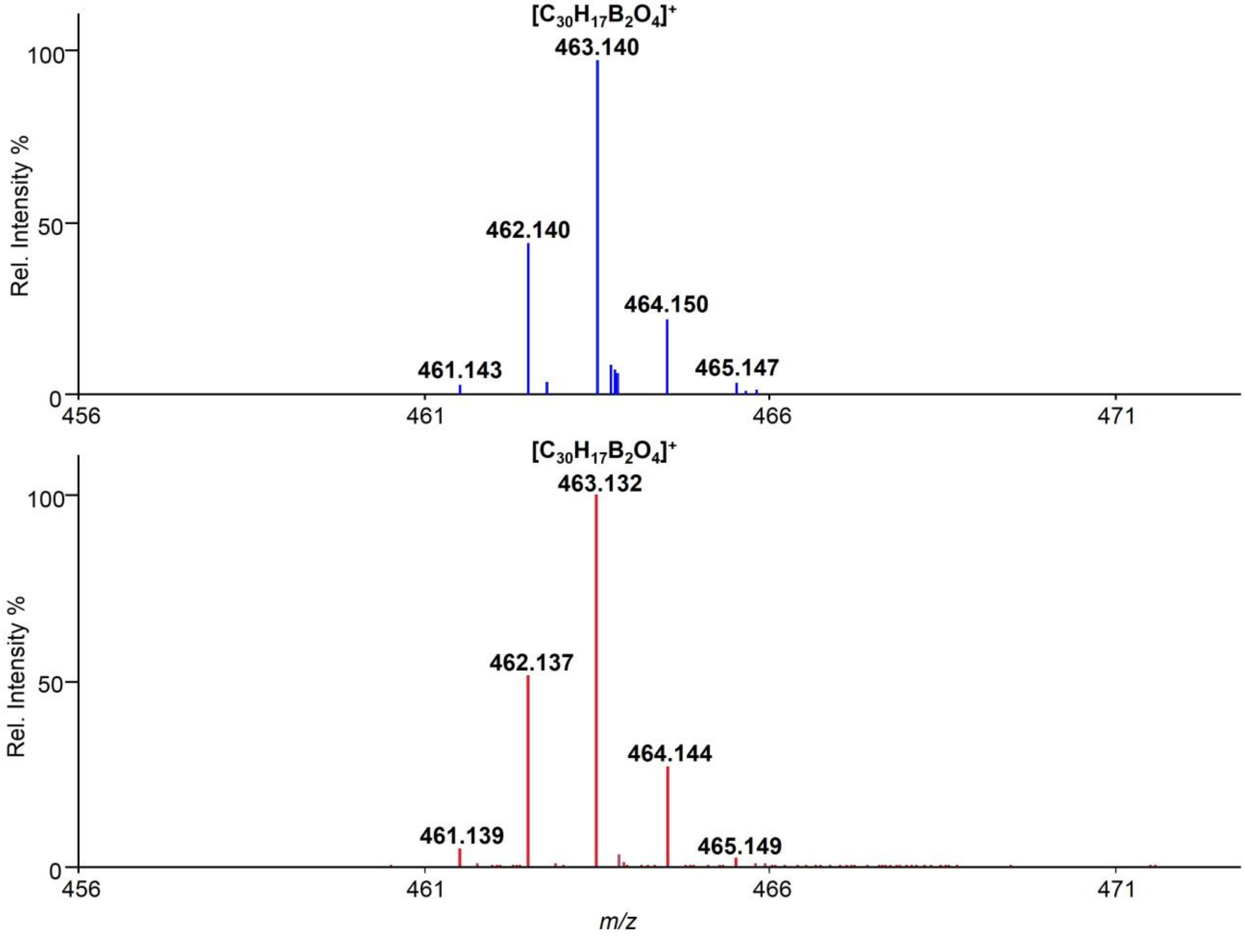

Figure S20. DART-MS spectra of **1** (bottom) and the quenched product of *in situ* generated $\mathbf{1}^{2-}$ (top). Mass spectra were acquired using a DART-SVP ion source (IonSense, Saugus, MA, USA) coupled to a JEOL AccuTOF time-offlight mass spectrometer (JEOL USA, Peabody, MA, USA). Settings: DART positive mode, 5–20 V (orifice voltage), 350 °C (heater temperature).

V. Crystal Structure Solution and Refinement Details

Data collection of **2**·2THF was performed on a Bruker D8 VENTURE X-ray diffractometer with PHOTON 100 CMOS shutterless mode detector equipped with a Cu-target X-ray tube (λ = 1.54178 Å) at T = 100(2) K. Data collection of **3**·0.5THF was performed on a Bruker D8 VENTURE X-ray diffractometer with PHOTON 100 CMOS detector equipped with a Mo-target X-ray tube (λ = 0.71073 Å) at T = 100(2) K. Data reduction and integration were performed with the Bruker software package SAINT (version 8.38A).[3] Data were corrected for absorption effects using the empirical methods as implemented in SADABS (version 2016/2).[4] The structure was solved by SHELXT (version 2015/5)[5] and refined by full-matrix least-squares procedures using the Bruker SHELXTL (version 2017/1)[6] software package. All non-hydrogen atoms were refined anisotropically. H-atoms were included at calculated positions and refined as riders, with U_{iso}(H) = 1.2 U_{eq}(C) and U_{iso}(H) = 1.5 U_{eq}(C) for methyl groups. In **2**·2THF, three THF molecules bound to a sodium cation were found to be disordered. The disorder was modelled with two orientations with relative occupancies of 0.62:0.38, 0.66:0.34 and 0.67:0.33 for the two parts separately. In each disordered THF molecule, the geometries of two disordered parts were restrained to be similar. The anisotropic displacement parameters of the disordered THF molecule in the direction of the bonds were restrained to be equal with a standard uncertainty of 0.004 Å^2. They were also restrained to have the same U_{ij} components, with a standard uncertainty of 0.01 Å^2. The structure model of **2**·2THF was refined as a 2-component inversion twin with BASF value refined to 0.35390. Two solvent THF molecules in **2**·2THF were refined anisotropically and their H-atoms were included at calculated positions and refined as riders, with U_{iso}(H) = 1.2 U_{eq}(C). In **3**·0.5THF, the 18-crown-6 ether molecule was found to be disordered and was modelled with two orientations having relative occupancies of 0.75:0.25 for the two parts. The geometries of the disordered parts were restrained to be similar. The anisotropic displacement parameters of the disordered molecules in the direction of the bonds were restrained to be equal with a standard uncertainty of 0.004 Å^2. They were also restrained to have the same U_{ij} components, with a standard uncertainty of 0.01 Å^2. In the unit cell of **3**·0.5THF, there is one-half THF solvent molecule that was found to be severely disordered and was removed by the SQUEEZE routine in PLATON (version 201117).[7] The total void volume was 159 Å^3 indicated by PLATON, equivalent to 5.12 % of the unit cell's total volume. Further crystal and data collection details are listed in Table S1.

Table S1. Crystal Data and Structure Refinement Parameters for **2**·2THF and **3**·0.5THF.

Compound	**2**·2THF	**3**·0.5THF
Empirical formula	$C_{78}H_{112}B_2Na_2O_{22}$	$C_{64}H_{84}B_2K_2O_{18.5}$
Formula weight	1469.28	1249.13
Temperature (K)	100(2)	100(2)
Wavelength (Å)	1.54178	0.71073
Crystal system	Orthorhombic	Triclinic
Space group	$Pna2_1$	*P*-1
a (Å)	18.0991 (6)	13.0199(11)
b (Å)	21.4983 (8)	13.0961(11)
c (Å)	19.8705 (7)	19.1830(15)
α (°)	90.00	76.781(2)
β (°)	90.00	79.297(2)
γ (°)	90.00	80.822(2)
V (Å^3)	7731.6 (5)	3105.3(4)
Z	4	2
ρ_{calcd} (g·cm^{-3})	1.262	1.336
μ (mm^{-1})	0.835	0.226
F(000)	3152	1328
Crystal size (mm)	0.02×0.04×0.09	0.03×0.21×0.25
θ range for data collection (°)	3.028-66.797	2.924-25.138
Reflections collected	195276	53886
Independent reflections	13697 [R_{int} = 0.2403]	11065 [R_{int} = 0.0782]
Transmission factors (min/max)	0.8742/1	0.8869/1
Data/restraints/params.	13697/661/1076	11065/883/920
$R1$,[a] $wR2$[b] ($I > 2\sigma(I)$)	0.0611, 0.1259	0.0613, 0.1042
$R1$,[a] $wR2$[b] (all data)	0.0950, 0.1419	0.1170, 0.1327
Quality-of-fit[c]	1.047	1.034

[a] $R1 = \Sigma||F_o|-|F_c||/\Sigma|F_o|$. [b] $wR2 = [\Sigma[w(F_o^2-F_c^2)^2]/\Sigma[w(F_o^2)^2]]$.
[c] Quality-of-fit = $[\Sigma[w(F_o^2-F_c^2)^2]/(N_{obs}-N_{params})]^{½}$, based on all data.

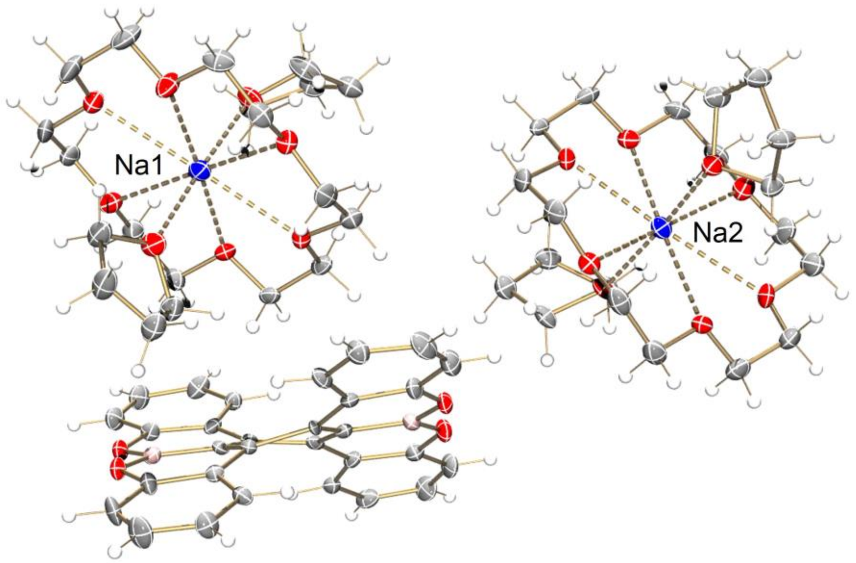

Figure S21. ORTEP drawing of asymmetric unit of **2**, drawn with thermal ellipsoids at the 40% probability level. Color scheme used: C grey, H white, O red, B pink and Na blue.

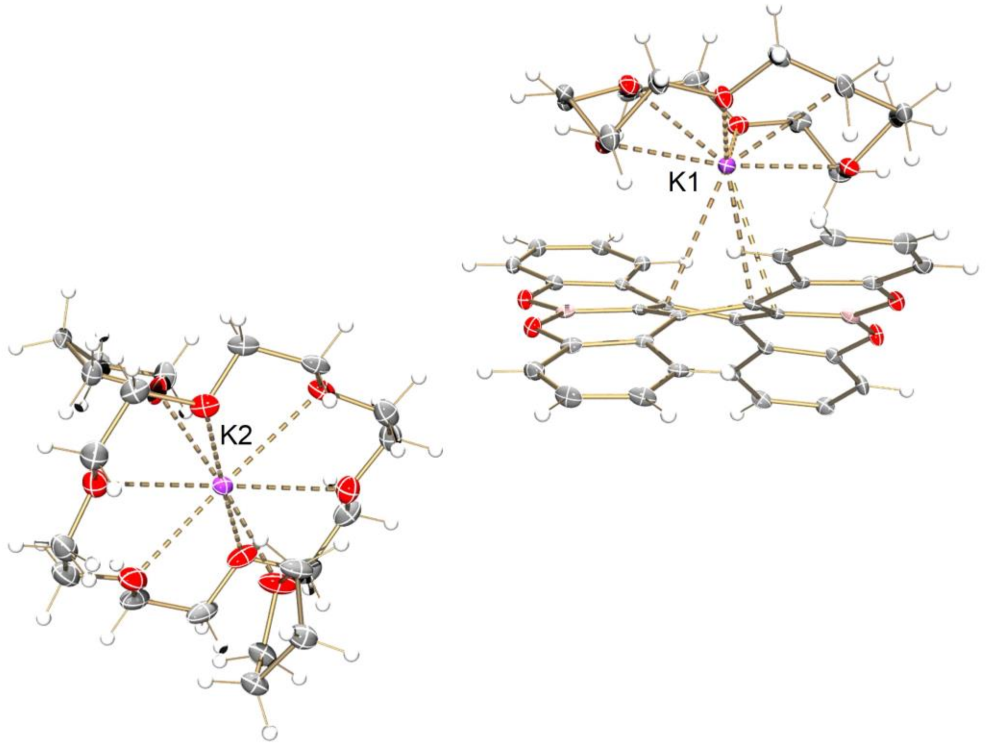

Figure S22. ORTEP drawing of asymmetric unit of **3**, drawn with thermal ellipsoids at the 40% probability level. Color scheme used: C grey, H white, O red, B pink and K dark orchid.

Table S2. Selected C–C bond distances in **1**, **2** and **3** (in Å) along with C-atom numbering scheme.

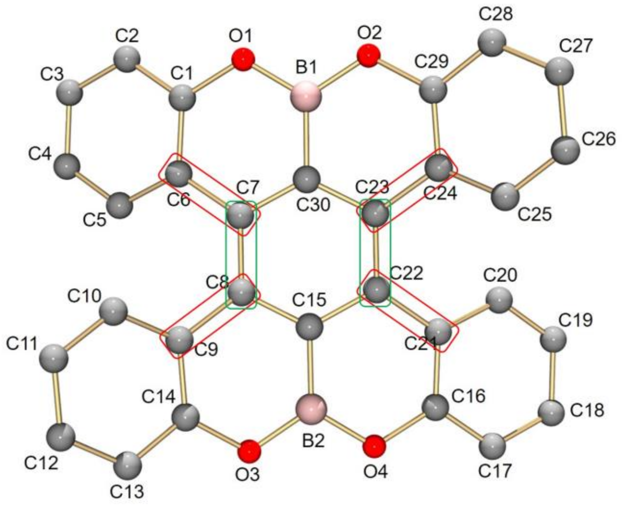

Bond	**1**	**2**	**3**	Bond	**1**	**2**	**3**
C1–C2	1.393(5)	1.365(9)	1.382(5)	C15–C22	1.403(5)	1.411(9)	1.412(4)
C1–C6	1.397(5)	1.415(9)	1.420(5)	C16–C17	1.385(5)	1.368(9)	1.375(5)
C2–C3	1.378(5)	1.398(9)	1.397(5)	C16–C21	1.411(5)	1.424(9)	1.425(5)
C3–C4	1.388(5)	1.394(10)	1.379(5)	C17–C18	1.383(5)	1.381(10)	1.397(5)
C4–C5	1.386(5)	1.378(9)	1.389(5)	C18–C19	1.393(5)	1.391(10)	1.383(5)
C5–C6	1.493(5)	1.417(8)	1.419(4)	C19–C20	1.381(5)	1.368(9)	1.376(5)
C6–C7	1.478(5)	1.446(9)	1.445(4)	C20–C21	1.404(5)	1.440(9)	1.421(4)
C7–C8	1.430(5)	1.475(9)	1.479(4)	C21–C22	1.472(5)	1.446(9)	1.448(4)
C7–C30	1.402(5)	1.401(9)	1.401(9)	C22–C23	1.430(5)	1.499(9)	1.483(4)
C8–C9	1.467(5)	1.450(9)	1.447(4)	C23–C24	1.474(5)	1.435(9)	1.440(4)
C8–C15	1.401(5)	1.408(9)	1.415(4)	C23–C30	1.416(5)	1.419(9)	1.420(4)
C9–C10	1.413(5)	1.404(9)	1.421(5)	C24–C25	1.415(5)	1.398(8)	1.398(8)
C9–C14	1.413(5)	1.428(9)	1.418(5)	C24–C29	1.406(5)	1.431(9)	1.423(5)
C10–C11	1.374(5)	1.398(9)	1.383(4)	C25–C26	1.368(5)	1.393(9)	1.380(5)
C11–C12	1.403(5)	1.392(10)	1.386(5)	C26–C27	1.396(5)	1.391(10)	1.386(5)
C12–C13	1.369(5)	1.387(10)	1.392(5)	C27–C28	1.371(5)	1.378(10)	1.389(5)
C13–C14	1.386(5)	1.369(9)	1.376(4)	C28–C29	1.376(5)	1.365(9)	1.376(5)

(Note: distance difference over 0.2 is labeled with green (higher) and red (lower) color)

Table S3. Selected bond distances in **1**, **2** and **3** (in Å).

Bond	**1**	**2**	**3**
B1–O1	1.377(5)	1.368(8)	1.378(4)
B1–O2	1.377(5)	1.375(8)	1.368(4)
B1–C30	1.512(6)	1.519(7)	1.510(5)
B2–O3	1.366(6)	1.374(9)	1.369(4)
B2–O4	1.370(6)	1.366(9)	1.383(4)
B2–C15	1.516(6)	1.516(7)	1.504(5)

Table S4. Selected torsion angles and plane angles in **1**, **2** and **3** (in °) along with a labeling scheme.

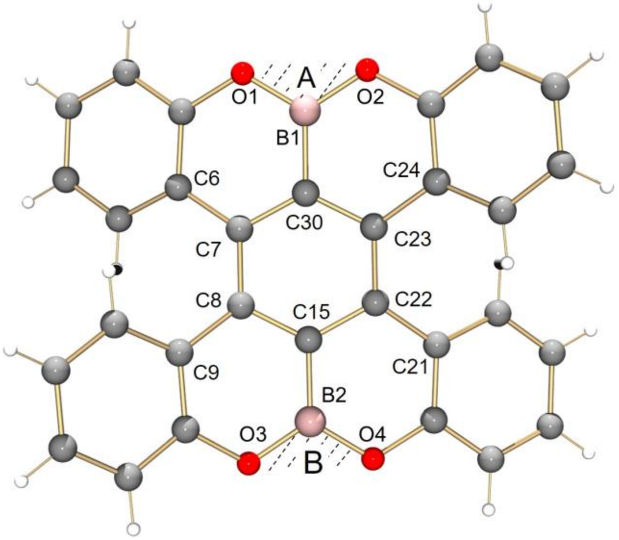

Name	**1**	**2**	**3**
C6−C7−C8−C9	32.3	34.19	34.05
C15−C8−C7−C30	24.5	19.44	21.67
C15−C22−C23−C30	23.54	18.95	20.92
C21−C22−C23−C24	28.2	35.06	35.08
A/B	21.39	24.65	31.75

VI. Theoretical Calculations

Density functional theory (DFT) calculations were performed using the Gaussian 09 software package.[8] The geometries were optimized and the energies were calculated at the B3LYP/6-311++G(d,p) level. No symmetry restrictions were used during the calculations. The calculated structures correspond to local energy minima (without imaginary frequencies). The electrostatic potential (ESP) maps of the neutral **1** and the dianionic **1**$^{2-}$ were generated by GaussView.

Table S5. Cartesian coordinates of the neutral **1**.

Tag	Symbol	X	Y	Z
1	C	-1.242338	0.696617	-0.144123
2	C	-1.242313	-0.696663	0.144067
3	C	0.000031	-1.353778	0.000026
4	C	1.242353	-0.696628	-0.144071
5	C	1.242328	0.696673	0.144023
6	C	-0.000017	1.353777	-0.000071
7	C	-2.372665	1.511491	-0.628220
8	C	-2.372605	-1.511569	0.628189
9	C	2.372674	-1.511538	-0.628128
10	C	2.372610	1.511616	0.628109
11	C	2.274136	-2.922169	-0.655305
12	C	3.315686	-3.720183	-1.128952
13	C	4.460349	-3.133527	-1.646196
14	C	4.554713	-1.740098	-1.708436
15	C	3.528233	-0.953085	-1.211809
16	C	-3.528196	-0.953101	1.211791
17	C	-4.554649	-1.740103	1.708490
18	C	-4.460224	-3.133535	1.646405
19	C	-3.315528	-3.720199	1.129242
20	C	-2.274006	-2.922192	0.655522
21	C	-2.274125	2.922120	-0.655513
22	C	-3.315684	3.720097	-1.129200
23	C	-4.460361	3.133401	-1.646368
24	C	-4.554732	1.739967	-1.708487
25	C	-3.528243	0.952992	-1.211819
26	C	3.528180	0.953194	1.211798
27	C	4.554622	1.740235	1.708457
28	C	4.460206	3.133661	1.646245
29	C	3.315525	3.720285	1.129005
30	C	2.274012	2.922241	0.655326

31	O	-1.144542	3.584586	-0.251034
32	O	1.144419	3.584644	0.250771
33	O	-1.144405	-3.584625	0.251036
34	O	1.144559	-3.584605	-0.250762
35	B	-0.000042	2.876053	-0.000139
36	B	0.000059	-2.876054	0.000081
37	H	3.187953	-4.795469	-1.102502
38	H	5.263970	-3.755949	-2.022322
39	H	5.425661	-1.270387	-2.150068
40	H	3.610699	0.122570	-1.282513
41	H	-3.610710	0.122560	1.282369
42	H	-5.425625	-1.270379	2.150053
43	H	-5.263824	-3.755950	2.022587
44	H	-3.187751	-4.795482	1.102906
45	H	-3.187949	4.795385	-1.102839
46	H	-5.263990	3.755795	-2.022526
47	H	-5.425695	1.270221	-2.150055
48	H	-3.610717	-0.122669	-1.282424
49	H	3.610686	-0.122461	1.282476
50	H	5.425582	1.270546	2.150091
51	H	5.263798	3.756106	2.022397
52	H	3.187751	4.795566	1.102579

Table S6. Cartesian coordinates of the dianion $\mathbf{1}^{2-}$.

Tag	Symbol	X	Y	Z
1	C	-1.247309	0.725717	-0.138211
2	C	-1.247302	-0.725729	0.138195
3	C	0.000004	-1.376035	0.000036
4	C	1.247305	-0.725726	-0.138162
5	C	1.247298	0.725738	0.138151
6	C	-0.000009	1.376035	-0.000050
7	C	-2.339475	1.506576	-0.666373
8	C	-2.339460	-1.506598	0.666361
9	C	2.339474	-1.506618	-0.666268
10	C	2.339456	1.506639	0.666265
11	C	2.253880	-2.934918	-0.756893
12	C	3.275168	-3.714186	-1.273562
13	C	4.452925	-3.126466	-1.765383
14	C	4.554181	-1.732701	-1.739763
15	C	3.533973	-0.948184	-1.214772

16	C	-3.533956	-0.948139	1.214846
17	C	-4.554150	-1.732631	1.739901
18	C	-4.452880	-3.126393	1.765610
19	C	-3.275124	-3.714134	1.273816
20	C	-2.253849	-2.934891	0.757083
21	C	-2.253881	2.934871	-0.757084
22	C	-3.275165	3.714107	-1.273809
23	C	-4.452917	3.126356	-1.765605
24	C	-4.554172	1.732593	-1.739904
25	C	-3.533968	0.948108	-1.214857
26	C	3.533957	0.948214	1.214775
27	C	4.554153	1.732739	1.739778
28	C	4.452882	3.126503	1.765405
29	C	3.275122	3.714213	1.273582
30	C	2.253845	2.934938	0.756901
31	O	-1.125793	3.611184	-0.331133
32	O	1.125751	3.611215	0.330907
33	O	-1.125756	-3.611195	0.331129
34	O	1.125789	-3.611205	-0.330907
35	B	-0.000015	2.889565	-0.000099
36	B	0.000010	-2.889565	0.000080
37	H	3.128208	-4.789621	-1.293442
38	H	5.250981	-3.742755	-2.167002
39	H	5.440289	-1.245283	-2.139168
40	H	3.634501	0.128127	-1.233055
41	H	-3.634493	0.128173	1.233061
42	H	-5.440258	-1.245195	2.139286
43	H	-5.250926	-3.742664	2.167279
44	H	-3.128152	-4.789567	1.293765
45	H	-3.128205	4.789541	-1.293752
46	H	-5.250970	3.742621	-2.167269
47	H	-5.440275	1.245150	-2.139289
48	H	-3.634494	-0.128204	-1.233078
49	H	3.634496	-0.128096	1.233052
50	H	5.440264	1.245328	2.139186
51	H	5.250930	3.742798	2.167032
52	H	3.128150	4.789647	1.293467

VII. References

[1] N. V. Kozhemyakina, J. Nuss, M. Jansen, *Z. Anorg. Allg. Chem.* **2009**, *635*, 1355-1361.

[2] X.-Y. Wang, A. Narita, W. Zhang, X. Feng, K. Müllen, *J. Am. Chem. Soc.* **2016**, *138*, 9021-9024.

[3] SAINT; part of Bruker APEX3 software package (version 2017.3-0): Bruker AXS, 2017.

[4] SADABS; part of Bruker APEX3 software package (version 2017.3-0): Bruker AXS, 2017.

[5] G. M. Sheldrick *Acta Cryst.* **2015**, *A71*, 3-8.

[6] G. M. Sheldrick, *Acta Cryst.* **2015**, *C71*, 3-8.

[7] A. L. Spek, *Acta Cryst.* **2015**, *C71*, 9-19.

[8] Gaussian 09, Revision D.01, Frisch, M. J.; Trucks, G. W.; Schlegel, H. B.; Scuseria, G. E.; Robb, M. A.; Cheeseman, J. R.; Scalmani, G.; Barone, V.; Mennucci, B.; Petersson, G. A.; Nakatsuji, H.; Caricato, M.; Li, X.; Hratchian, H. P.; Izmaylov, A. F.; Bloino, J.; Zheng, G.; Sonnenberg, J. L.; Hada, M.; Ehara, M.; Toyota, K.; Fukuda, R.; Hasegawa, J.; Ishida, M.; Nakajima, T.; Honda, Y.; Kitao, O.; Nakai, H.; Vreven, T.; Montgomery, Jr., J. A.; Peralta, J. E.; Ogliaro, F.; Bearpark, M.; Heyd, J. J.; Brothers, E.; Kudin, K. N.; Staroverov, V. N.; Kobayashi, R.; Normand, J.; Raghavachari, K.; Rendell, A.; Burant, J. C.; Iyengar, S. S.; Tomasi, J.; Cossi, M.;Rega, N.; Millam, N. J.; Klene, M.; Knox, J. E.; Cross, J. B.; Bakken, V.; Adamo, C.; Jaramillo, J.; Gomperts, R.; Stratmann, R. E.; Yazyev, O.; Austin, A. J.; Cammi, R.; Pomelli, C.; Ochterski, J. W.; Martin, R. L.; Morokuma, K.; Zakrzewski, V. G.; Voth, G. A.; Salvador, P.; Dannenberg, J. J.; Dapprich, S.; Daniels, A. D.; Farkas, Ö.; Foresman, J. B.; Ortiz, J. V.; Cioslowski, J.; Fox, D. J. Gaussian, Inc., Wallingford CT, 2013.
